# Linkage map construction and QTL mapping for morphological traits in *Ipomoea trifida*, a diploid sweetpotato relative

**DOI:** 10.1002/tpg2.70106

**Published:** 2025-09-18

**Authors:** Ana Paula Alves da Mata, Dorcus C. Gemenet, Federico Diaz, Maria David, Veronica Mosquera, João Ricardo Bachega Feijó Rosa, Iara Gonçalves dos Santos, Gabriel de Siqueira Gesteira, Marcelo Mollinari, Awais Khan, G. Craig Yencho, Zhao‐Bang Zeng, Guilherme da Silva Pereira

**Affiliations:** ^1^ Department of Agronomy Federal University of Viçosa Viçosa Minas Gerais Brazil; ^2^ International Maize and Wheat Improvement Center (CIMMYT) Nairobi Kenya; ^3^ International Potato Center (CIP) Lima Peru; ^4^ Department of Horticultural Science North Carolina State University Raleigh North Carolina USA; ^5^ School of Integrative Plant Science Cornell University Geneva New York USA

## Abstract

*Ipomoea trifida* G. Don (2*n* = 2*x* = 30) is considered the closest known diploid relative and a wild ancestor of the autohexaploid sweetpotato, *Ipomoea batatas* (L.) Lam. (2*n* = 6*x* = 90). This study aimed to map quantitative trait loci (QTLs) in a diploid full‐sib population (M9 × M19) consisting of 210 progenies based on a high‐density genetic linkage map constructed with single‐nucleotide polymorphisms (SNPs). In a randomized complete block design with four replications, the phenotypic evaluation of 11 morphological traits was conducted for 188 individuals in 2016 at the International Potato Center under screenhouse conditions in San Ramón, Peru. Heritabilities ranged from 0.30 to 0.80, and genetic correlations varied from −0.22 to 1. An integrated genetic map was constructed with 15 linkage groups and 6410 SNPs spanning 2440.47 cM using the *Onemap* v.3.0 R package. Major misassemblies were identified and properly fixed on chromosomes 2, 3, and 7. QTL mapping was performed using the composite interval mapping approach for each trait with *fullsibQTL* v.0.0.901 R package. A total of 37 QTLs were identified, with up to 42.39% of the proportion of phenotypic variance explained by a major QTL on chromosome 3 for a leaf shape‐related trait. Reference genome refining and QTL‐linked markers contribute to advancing genetic and genomic research on *I. trifida* and may support sweetpotato breeding programs targeting ornamental traits.

## Plain Language Summary

Sweetpotato (*Ipomoea batatas*) is an important food and ornamental polyploid crop with a complex genome. Its closest wild, diploid relative, *Ipomoea trifida*, emerges as a relatively simpler model for conducting genetic studies. Understanding the genetic basis of morphological traits in *I. trifida* can provide essential insights for improving agricultural and ornamental sweetpotato. We aimed to study the genetic architecture of morphological traits in an *I. trifida* full‐sib family. Using a high‐density genetic linkage map, we were able to unravel and fix major reference genome misassemblies in chromosomes 2, 3, and 7. In addition, 37 quantitative trait loci (QTLs) for eleven morphological traits were identified, including major QTL for leaf morphological traits. These findings advance the genomic characterization of the *Ipomoea* genus, offering support for future efforts to improve agricultural and ornamental traits in a globally relevant crop such as sweetpotato.

**Abbreviations**
BLUP: best linear unbiased prediction
CIM: composite interval mapping
CIP: International Potato Center
GBS: genotyping‐by‐sequencing
LA: leaf area
LG: linkage group
LOD: logarithm of the odds
QTL: quantitative trait locus
SNP: single‐nucleotide polymorphism
UPGMA: unweighted pair group method with arithmetic mean
WGD: whole‐genome duplication

## 1 INTRODUCTION

*Ipomoea batatas* (L.) Lam. (2*n* = 6*x* = 90), commonly known as sweetpotato, is a globally significant crop, particularly in tropical and subtropical regions, where it plays a crucial role in food security. Despite its importance, advancements in understanding the genetics of this species have been challenging, primarily due to its complex polyploid nature (Austin, 1988). This genomic complexity poses a significant obstacle to deciphering genetic and genomic patterns and trait variation (Li et al., 2019; S. Wu et al., 2018).

Sweetpotato has emerged as a critical pillar in the fight against food insecurity aggravated by climate change. Its capacity to survive in challenging circumstances such as poor soil conditions and locations susceptible to frequent and unpredictable droughts makes it a resilient and reliable crop. Its storage roots have high nutritional value, including vitamins, minerals, and carbohydrates, providing an excellent long‐term sustenance source (Chen et al., 2019; Hong et al., 2022; Liu et al., 2022; S. Wu et al., 2018). At the same time, its culinary diversity and high preservation capacity make it an important resource in communities with limited food sources. In addition, the leaves can also be animal and human food sources, the roots can be used to manufacture food products and in the cosmetics industry (Hong et al., 2022; Tang et al., 2023), and the plants may constitute ornamental gardens, using varieties with lush leaves, different brightness, colors, and shapes (de Sousa et al., 2018; Fischer et al., 2015; Jackson et al., 2020; Tamura et al., 2024).

Domesticated around 5000 years ago between Central and South America (Austin, 1988), sweetpotato was taken to European and African continents at the time of the great navigations, at the beginning of the 16th century (Roullier et al., 2013). Around 800,000 and 500,000 years ago, two recent sweetpotato whole‐genome duplication (WGD) events occurred (Muñoz‐Rodríguez et al., 2018; J. Yang et al., 2017). In the first WGD, the tetraploid progenitor of *I. batatas* was produced. After a cross between this tetraploid progenitor and a diploid progenitor, the second WGD occurred, giving rise to the modern cultivated *I. batatas* (J. Yang et al., 2017). Although the tetraploid progenitor is still unknown, according to several molecular studies, the most likely diploid progenitor of the hexaploid sweetpotato is *Ipomoea trifida* G. Don (Huang et al., 2002; Muñoz‐Rodríguez et al., 2018; Rajapakse et al., 2004; Roullier et al., 2013; Srisuwan et al., 2006).

*I. trifida* (2*n* = 2*x* = 30) is considered the closest known diploid relative and a putative wild ancestor of the hexaploid sweetpotato, and it is part of a polyploidy complex ranging from diploid to hexaploid (Nishiyama et al., 1975; Shiotani & Kawase, 1989). Although recent phylogenomic analyses have suggested that certain tetraploid relatives such as *Ipomoea aequatoriensis* may be more directly involved in the origin of cultivated sweetpotato (Yan et al., 2024), *I. trifida* remains a widely accepted model species due to its botanical similarity to hexaploid sweetpotato and its smaller diploid genome, which has been sequenced and extensively used as a reference for hexaploid sweetpotato (Hirakawa et al., 2015; Li et al., 2022, 2019; Nishiyama, 1971; S. Wu et al., 2018; Z. Yang et al., 2019). Importantly, *I. trifida* is predominantly an outcrossing species, as it possesses a sporophytic self‐incompatibility system that promotes cross‐pollination in natural populations (Kowyama et al., 1994; Tsuchiya, 2014). In 2018, S. Wu et al. (2018) published high‐quality genomic sequences of *I. trifida* and *Ipomoea triloba*, the latter also considered a putative ancestor, marking a significant breakthrough in the genetic and genomic studies of cultivated sweetpotato.

Advancements in genomic tools, such as quantitative trait loci (QTLs) mapping, are providing deeper insights into the relationship between the sweetpotato genome and its phenotypic expression. QTLs are regions of the chromosome that contribute to the variation of traits of interest. QTL mapping makes it possible to estimate the location, the genetic effects of such regions on a specific trait, and their contributions to total phenotypic variance. Among the available methods used for QTL mapping, composite interval mapping (CIM) (Zeng, 1994) stands out as it combines information from flanking markers (cofactors) to improve mapping accuracy. Gazaffi et al. (2014) extended CIM for outcrossing plant species, such as *I. trifida*, enabling QTL mapping based on a full‐sib population.

This study aimed to map QTL for morphological traits in a full‐sib population of *I. trifida*, consisting of 210 full‐sib progenies (M9 × M19), based on a high‐density genetic linkage map using single‐nucleotide polymorphism (SNP) markers derived from the genotyping‐by‐sequencing (GBS) methodology. The results obtained in this study can be applied to sweetpotato breeding programs, after proper marker validation, to accelerate selection decisions, especially on the development of new ornamental product profiles.

## 2 MATERIALS AND METHODS

### 2.1 Experimental data and phenotypic analysis

The mapping population was previously described by S. Wu et al. (2018). In brief, a full‐sib population consisting of 210 progenies, derived from the M9 × M19 cross (named CIP113735, where CIP stands for International Potato Center), was established and phenotyped at CIP. M9 (CIP107665.9) and M19 (CIP107665.19) are full‐sibs derived from a cross between the accessions DLP4653 (CIP460410) and DLP4597 (CIP460377) (Figure S1). M9 and M19 were selected to become parents due to their ability to form storage roots and relative molecular genetic distance as shown via microsatellite markers (A. Khan, personal communication, 2014).

A sample of 188 individuals, including both parents, was evaluated in San Ramón, Peru, in 2016, under screenhouse conditions using a randomized complete block design with four replications. Each replication included one cutting per genotype for a total of 186 full‐sibs and the two parents, all grown in 4‐L pots. Following the sweetpotato Crop Ontology (https://cropontology.org/term/CO_331:ROOT, Supporting Information S1) and previously published scales (CIP, AVRDC, IBPGR, 1991; Figure S2), the evaluated traits were as follows: leaf general outline (LEAFSHAP1, CO_331:0000021) in a 1–7 scale; leaf lobes type (LEAFSHAP2, CO_331:0000024) in a 0–9 scale; leaf lobe number (LLN) (LEAFSHAP3, CO_331:0000027) in a 1–9 scale; leaf central lobe shape (LEAFSHAP4, CO_331:0000030) in a 0–9 scale; number of leaves per plant (CO_331:0000673) measured at 12 days (LEFTPP_TP1), 30 days (LEFTPP_TP2), and 58–62 days (LEFTPP_TP3); plant height (CO_331:0000950) measured at 11 days (PLANTH_TP1) and 30 days (PLANTH_TP2) in centimeter; vine internode diameter (VINDIA, CO_331:0002031) in millimeter; and leaf area (LA) in square millimeter. LA was computed based on six representative leaves photographed against a black background and analyzed using the free software ImageJ (https://imagej.net/ij/).

A mixed model was used to obtain the best linear unbiased predictions (BLUPs)‐based adjusted means for individuals:

(1) $$y_{ij}=\mu +b_{j}+g_{i}+e_{ij}$$

where $y_{ij}$ is the phenotype of the ith genotype in jth block, μ is the overall mean, $b_{j}$ is the fixed effect of the jth block, $g_{i}$ is the random effect of the ith genotype where $g_{i}∼N\left(0,\sigma_{g}^{2}\right)$ and $\sigma_{g}^{2}$ is the genetic variance, and $e_{ij}$ is the random error, where $e_{ij}∼N\left(0,\sigma_{e}^{2}\right)$ and $\sigma_{e}^{2}$ is the residual variance.

Variance components were estimated via restricted maximum likelihood, and the significance of the genotypic effect was verified using the likelihood ratio test, assuming 5% probability. The BLUP‐derived adjusted means were extracted for further QTL mapping analyses. All analyses were carried out using the R package sommer v.4.1.2 (Covarrubias‐Pazaran, 2018). Broad‐sense heritability was calculated for each trait as follows:

(2) $$H^{2}=\frac{\sigma_{g}^{2}}{\sigma_{g}^{2}+\frac{\sigma_{e}^{2}}{r}}$$

where r is the number of blocks.

Pearson's correlations were obtained and tested, assuming a significance global level of 5%. Plots were generated using the R package ggplot2 v. 3.5.0 (Wickham, 2016).

### 2.2 Genotypic analysis

#### 2.2.1 Genetic map

A total of 26,968 SNP markers from the GBS methodology (S. Wu et al., 2018) were obtained for the 210 full‐sib progenies and their parents (M9 and M19). In brief, sequence reads were demultiplexed and barcodes were removed using the cutadapt v.3.1 software (Martin, 2011), followed by a quality control analysis using FastQC (available at https://www.bioinformatics.babraham.ac.uk/projects/fastqc/), FastQ Screen (Wingett & Andrews, 2018), and MultiQC (Ewels et al., 2016). The trimmed sequence reads were aligned to the *I. trifida* v.3.0 reference genome (S. Wu et al., 2018) using the bowtie2 v.2.1.0 software (Langmead & Salzberg, 2012), with the “–very‐sensitive‐local” flag to ensure restrictive alignments. The mapped reads were used as input for variant discovery with the GATK v.4.1.6 software (McKenna et al., 2010), according to the best practices recommendations (Van der Auwera et al., 2013), while skipping the removal of duplicates and the recalibration of bases and variants. The final set of variants was subject to hard‐filtering steps to ensure that genotypes were estimated on at least six reads and variants had an average read depth equal to or higher than 6*x* while keeping biallelic variants only.

After reading the variant call format file onto *Onemap* v.3.0 R package (Margarido et al., 2007), markers were subjected to additional filtering, that is, markers were filtered out due to missing information for either or both parents, and given a 20% and 50% missing data threshold for markers and individuals, respectively. Using the filtered data, the segregation of SNP markers was verified using a chi‐square test, followed by a Bonferroni correction, with a global significance level of 5%. This analysis determined whether the markers exhibited the expected segregation ratios of 1:1 (resulting from crosses of types D1.10, *aa* × *ab*, or D2.15, *ab* × *aa*) or 1:2:1 (type B3.7, *ab* × *ab*), depending on the parental genotypes for biallelic markers in a full‐sib population (R. Wu et al., 2002).

For constructing the linkage map, recombination fractions and linkage phases were estimated simultaneously for each pair of markers. Subsequently, linkage groups (LGs) were defined using two approaches: (i) based on the *I. trifida* reference genome, which considered the markers’ physical positions, and (ii) de novo, based on estimates of recombination fractions by employing the unweighted pair group method with arithmetic mean (UPGMA). The grouping obtained from the latter approach was then compared to the genomic information provided by the reference genome.

Ordering of markers was initially based on the *I. trifida* reference genome. Major misassemblies, when present, were assessed via recombination fraction heatmaps and manually fixed. LGs whose gaps were larger than 10 cM between markers were split into subgroups for subsequent manual correction. Subgroups containing up to 10 markers and markers originating from different chromosomes were submitted to the “try_seq” function. This function tests each unplaced marker at all possible locations along a given LG and assigns it to the position that yields the highest likelihood without changing the order of the markers initially placed on the LGs.

For the final map estimation, a global error rate of 0.15 was applied within the hidden Markov model. Genetic distances were obtained using the Kosambi mapping function (Kosambi, 1943). The visualization of the final map was obtained via the R package LinkageMapView v.2.1.2 (Ouellette et al., 2018).

#### 2.2.2 QTL analyses

CIM was conducted using the constructed linkage map and the adjusted means for each trait. The linkage map was scanned at 1‐cM intervals to calculate the multipoint probabilities of QTL genotypes, conditioned on the markers positioned along the map.

The linear model of QTL mapping was described by Gazaffi et al. (2014) as follows:

(3) $$y_{j}=Z_{j}\;\gamma +\alpha_{p}^{*}x_{pj}^{*}+\alpha_{q}^{*}x_{qj}^{*}+\delta_{pq}^{*}x_{pj}^{*}x_{qj}^{*}+\varepsilon_{j}$$

where $y_{j}$ is the phenotype of the jth individual (j = 1, 2, …, 𝑛), $Z_{j}$ is the matrix of the jth row, $\alpha_{p}$ and $\alpha_{q}$ are the additive effects of the QTL for the respective parents, $x_{pj}^{*}$ and $x_{qj}^{*}$ are the respective indicator variables, $\delta_{pq}$ is the intra‐locus interaction effect (dominance effect), and $\varepsilon_{j}$ $∼\;N(0,\sigma_{\varepsilon }^{2})$ is the error. γ is the vector (1+3c)×1 containing the intercept μ and c coefficients of the multiple linear regression parameters ($\alpha_{p},\alpha_{q}$, and $\delta_{pq}$) for each cofactor.

To account for the effects of QTL located outside the mapping region, up to 10 markers were used as cofactors in the model. Cofactors were selected and fixed across the genome for each trait using the Akaike information criterion (Akaike, 1973). Markers within a window size of 20 cM were not fitted as cofactors while searching for QTL within that interval.

A total of 1000 permutations were conducted to establish the QTL detection threshold at a 5% significance level. Once the number and positions of the QTL were identified, each was characterized based on the peak locations, including their significant effects, segregation patterns, and linkage phases with the flanking markers. QTL analyses were carried out using the R package *fullsibQTL* v.0.0.901 (Gazaffi et al., 2014).

Additionally, within 500 kb around major QTL peaks, a putative gene search was conducted in Sweetpotato Genomic Resources (https://sweetpotato.uga.edu/), using the *I. trifida* v.3.0 reference genome JBrowse, under the Representative HC Gene Models category. Gene annotations within these regions were checked to identify potential matches with information available in the published scientific literature.

## 3 RESULTS AND DISCUSSION

### 3.1 Phenotypic analysis

**FIGURE 1:**
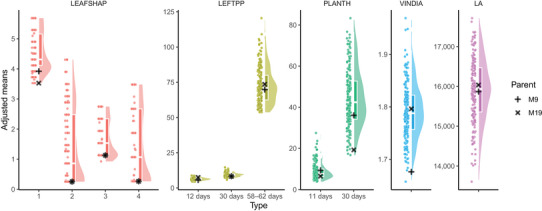
Distribution of adjusted means for the 11 evaluated traits in a full‐sib population of *Ipomoea trifida*: four different leaf shape traits (LEAFSHAP 1 through 4, in respective scales), number of leaves per plant (LEFTPP) across three different times (12, 30, and 58–62 days), plant height (PLANTH, in cm) across two different times (11 and 30 days), vine internode diameter (VINDIA, in mm), and leaf area (LA, in mm^2^).

From the mixed model analysis, it was possible to obtain adjusted means, genetic variances, and broad‐sense heritabilities for each trait (Table 1). Heritabilities ranged from 0.30 to 0.80. The traits with the highest heritability estimates were LEAFSHAP2, LEAFSHAP3, and LEAFSHAP4, while the lowest estimate was obtained for LEFTPP_TP3. The distribution of adjusted means of 188 individuals for each trait (Figure 1) has shown broad variation for all traits despite some similar phenotypes between parents. That points out one interesting feature of full‐sib populations derived from heterozygous parents (i.e., non‐inbreds). If a major QTL *Q* is fixed in each parent, let us say *QQ* in M9 and *qq* in M19, the full‐sibs will not segregate as all individuals will be *Qq*, despite M9 and M19 showing contrasting phenotypes. On the other hand, if the parental phenotypes are similar because parents carry a *Qq* genotype each, then segregation is expected. In fact, that seemed to be the case for leaf shape‐related traits (Table 1, Figure S3).

**TABLE 1:** Means for M9 and M19 parents, for the full‐sib population of *Ipomoea trifida*, and genetic variances ($\sigma_{g}^{2}$) and heritabilities ($H^{2}$) for morphological traits evaluated in 2016.

Trait	M9	M19	Full‐sibs	$\sigma_{g}^{2}$	$H^{2}$
Leaf general outline (LEAFSHAP1; Scale 1–7)	3.91	3.52	4.52	0.5378***	0.78
Leaf lobes type (LEAFSHAP2; Scale 0–9)	0.25	0.25	1.33	1.9528***	0.80
Leaf lobe number (LEAFSHAP3; Scale 1–9)	1.13	1.13	1.68	0.4534***	0.80
Leaf central lobe shape (LEAFSHAP4; Scale 0–9)	0.27	0.27	1.34	1.7510***	0.80
Number of leaves per plant measured at 12 days (LEFTPP_TP1; count)	5.67	7.55	6.44	1.7035***	0.53
Number of leaves per plant measured at 30 days (LEFTPP_TP2; count)	8.40	8.25	9.37	3.2614***	0.59
Number of leaves per plant measured at 58–62 days (LEFTPP_TP3; count)	69.81	73.57	72.74	545.6088***	0.30
Plant height measured at 11 days (PLANTH_TP1; cm)	9.35	6.52	8.85	24.5811***	0.64
Plant height measured at 30 days (PLANTH_TP2; cm)	36.11	19.29	44.21	259.2985***	0.74
Vine internode diameter (VINDIA; mm)	1.68	1.80	1.79	0.0078***	0.37
Leaf area (LA; mm^2^)	15,867.19	16,032.84	15,893.23	1,516,030***	0.41

*Note*: Statistical significance for the genetic variance was tested using the likelihood ratio test (LRT; **p < 0.01, ***p < 0.001). Trait measurement units are indicated in parentheses.

Devarajan et al. (2013) conducted a study using 22 sweetpotato genotypes to estimate the variability in morphology, yield, yield components, and other traits. They evaluated 22 traits, including leaf general outline, leaf central lobe shape, and mature leaf size, which showed heritabilities of 1.00, 1.00, and 0.26, respectively. Despite differences in species and population sizes between studies, their findings partially align with ours. Specifically, for LEAFSHAP1 and LEAFSHAP4, heritabilities were 0.78 and 0.80.

**FIGURE 2:**
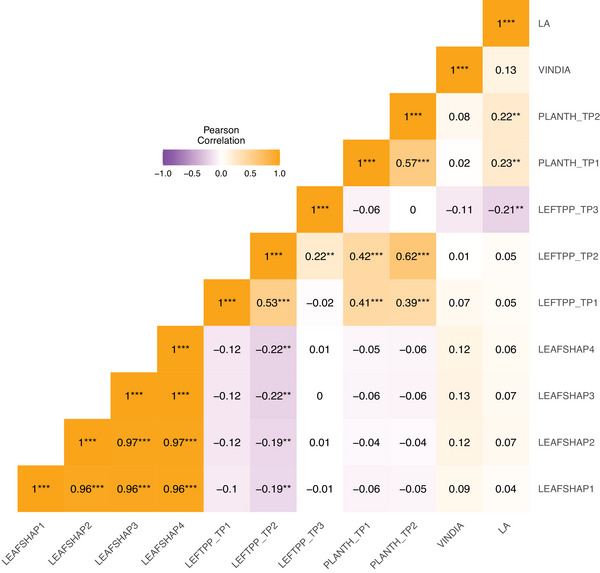
Pearson's correlation among adjusted means for leaf general outline (LEAFSHAP1); leaf lobes type (LEAFSHAP2); leaf lobe number (LEAFSHAP3); leaf central lobe shape (LEAFSHAP4); number of leaves per plant measured at 12 days (LEFTPP_TP1), at 30 days (LEFTPP_TP2), and at 58–62 days (LEFTPP_TP3); plant height measured at 11 days (PLANTH_TP1) and at 30 days (PLANTH_TP2); vine internode diameter (VINDIA); and leaf area (LA). **p < 0.01, ***p < 0.001.

Pairwise trait correlations between traits adjusted means ranged from −0.22 to 1 (Figure 2). LEAFSHAP1, LEAFSHAP2, LEAFSHAP3, and LEAFSHAP4 have shown high correlations among them (0.96–1). LEFTPP_TP1 and LEFTPP_TP2 have shown negative correlations with LEAFSHAP1 (−0.10 and −0.19), LEAFSHAP2 (−0.12 and −0.19), LEAFSHAP3 (−0.12 and −0.22), and LEAFSHAP4 (−0.12 and −0.22), whereas the correlations between them and LEFTPP_TP3 were around 0 (−0.01 to 0.01). PLANTH_TP1 and PLANTH_TP2 also had nonsignificant correlations with LEAFSHAP1, LEAFSHAP2, LEAFSHAP3, and LEAFSHAP4 (−0.06 to −0.04). PLANTH_TP1 and PLANTH_TP2 had high and positive correlations with LEFTPP_TP1 and LEFTPP_TP2 but nonsignificant ones with LEFTPP_TP3 (−0.06 and 0). VINDIA and LA had weak, nonsignificant correlations with LEAFSHAP1 (0.09 and 0.04), LEAFSHAP2 (0.12 and 0.07), LEAFSHAP3 (0.13 and 0.07), and LEAFSHAP4 (0.12 and 0.06), whereas LA had positive correlations with PLANTH_TP1 and PLANTH_TP2 (0.23 and 0.22), and a negative correlation with LEFTPP_TP3 (−0.21). The negative correlation between leaf shape traits (LEAFSHAP1–LEAFSHAP4) and LEFTPP_TP2, as well as between LA and LEFTPP_TP3, suggests a trade‐off in resource allocation, where plants with larger or more complex leaves produce fewer leaves overall (Ma et al., 2025; Scott & Aarssen, 2012; Whitman & Aarssen, 2010; D. Yang et al., 2008).

### 3.2 Genetic map

Initially, the dataset included 210 individuals, and 26,968 markers distributed among three segregation types according to R. Wu et al. (2002): B3.7 (10,255), D1.10 (6048), and D2.15 (5011). These types correspond to different expected segregation patterns in full‐sib populations, with B3.7 representing *ab × ab* crosses (1:2:1), D1.10 representing *ab × aa* (1:1), and D2.15 representing *aa × ab* (1:1). The *Onemap* R package removed 2767 markers due to missing parental genotype information, and 4349 markers were excluded because both parents were homozygous, resulting in non‐informative markers. A total of 19,852 markers remained from this first data filtering.

Next, a 20% missing data threshold was applied, eliminating 12,984 markers and leaving 8330 markers for further analysis, distributed as follows: B3.7 (4537), D1.10 (1943), and D2.15 (1850). Filtering for individuals with more than 50% missing data resulted in the removal of 11 individuals, reducing the sample size from 210 to 199. After applying a chi‐square test (with a global significance level of 5% using Bonferroni correction) to assess marker segregation among the filtered markers, 22% showed segregation distortion. Ultimately, 6464 non‐distorted, high‐quality markers remained for analysis.

**FIGURE 3:**
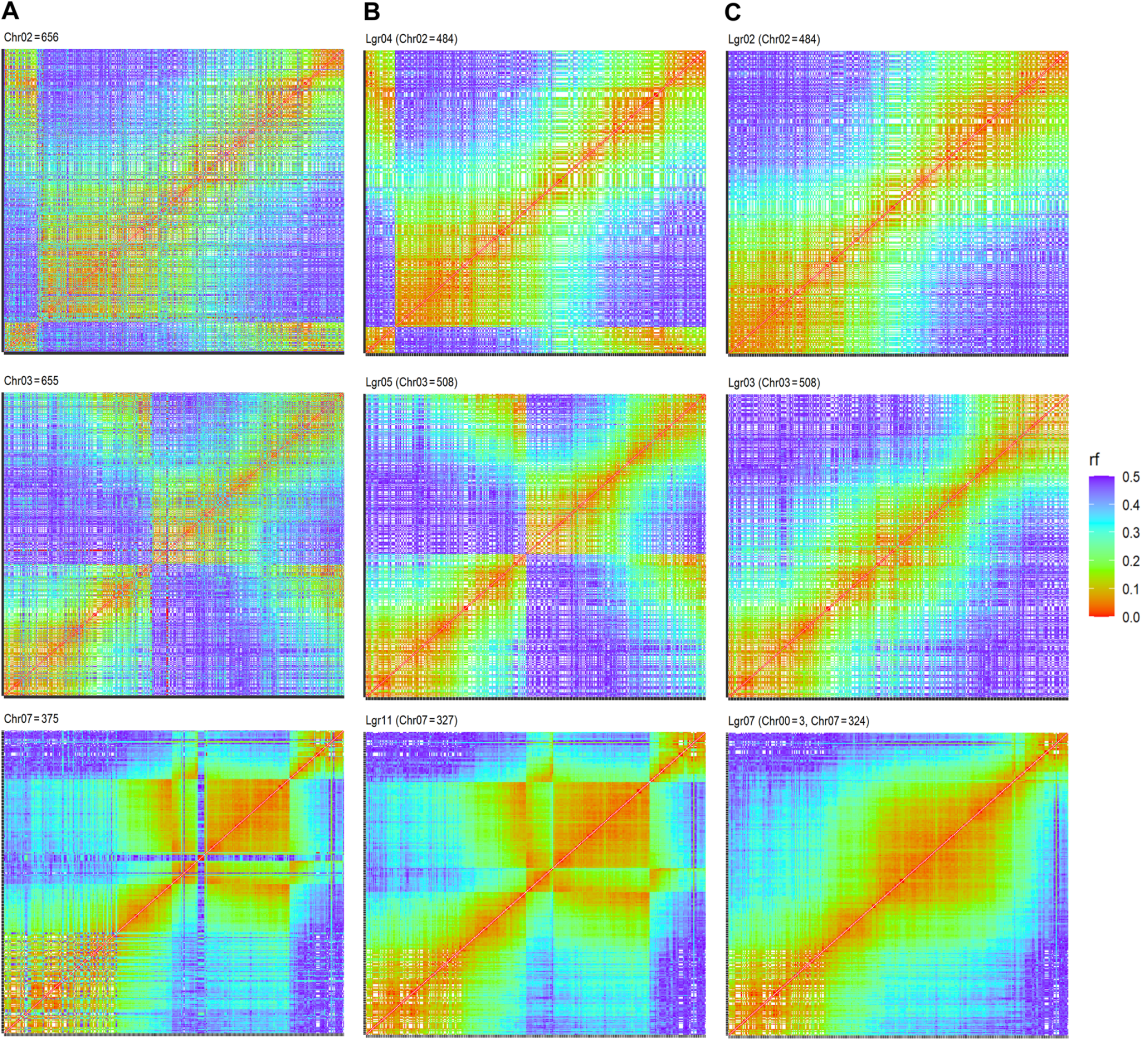
Heatmaps of recombination fractions (rf) between markers from chromosomes (Chrs) 2 (top row), 3 (middle row), and 7 (bottom row). (A) Original genome‐based grouping and ordering, (B) de novo UPGMA (unweighted pair group method with arithmetic mean)‐based grouping and genome ordering, and (C) de novo UPGMA‐based grouping and manual correction of major misassemblies.

During the analysis of heatmaps from two‐point recombination fractions, and leveraging the grouping and ordering provided by the reference genome, major misassemblies were identified in three out of the 15 chromosomes, namely 2, 3, and 7 (Figure 3a, Figure S4). These misassemblies were identified through off‐diagonal patterns in the heatmaps, which indicated presumed adjacent markers to be, in fact, genetically distant. Additionally, LGs split due to gaps larger than 10 cM, resulting in 30 subgroups, when a minor misassembly on chromosome 6 was identified and manually fixed.

While constructing the first sweetpotato genetic map using SNPs, Mollinari et al. (2020) analyzed a population of 315 individuals derived from a cross between the Beauregard and Tanzania varieties. The authors highlighted the high collinearity between the reference genomes of *I. trifida* and *I. triloba* (S. Wu et al., 2018) and that of the sweetpotato. They also identified putative chromosomal inversions in LGs 2, 3, 6, and 7, which they attributed to unique structural modifications in sweetpotato. However, these putative inversions were found in our study to be an artifact due to misassemblies.

**FIGURE 4:**
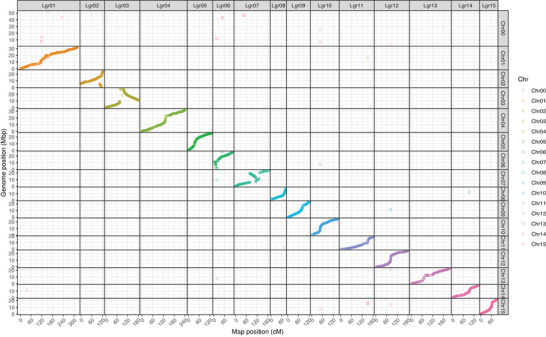
Markers positioned along linkage groups (*x*‐axis, in centiMorgans) and chromosomes (Chrs) (*y*‐axis, in megabase pairs).

Some LGs initially contained markers assigned to different chromosomes, as detected by UPGMA. This method grouped markers based on recombination frequency (de novo), and inconsistent markers were subsequently removed (Figure 3b, Figure S5). For better visualization and clarity, Figure 4 illustrates the original positions of the markers on the chromosomes alongside their corrected positions in the LGs.

Markers originally assigned to different chromosomes (as detected by UPGMA) and markers from the same chromosome that were not incorporated during the manual reordering were analyzed to determine their optimal positions (Figure 3c, Figure S6). The “try_seq” function was applied to evaluate each unplaced marker across all possible intervals within its corresponding LG, and to assign it to the position with the highest likelihood.

In addition, 32 markers were fixed, where 16 markers were likely originally misassigned to other chromosomes, and 16 other markers were previously unassigned to any chromosome (Chr00). Here, they were positioned as follows: five on chromosome 1, five on chromosome 6, three on chromosome 7, two on chromosome 10, and one on chromosome 11 (Table 2).

**TABLE 2:** Markers on each chromosome (Chr) and markers assigned to each one that were previously incorrectly assigned to other chromosomes.

Chromosomes	Number of markers	Origin and number of markers fixed locations	Size (cM)	Max gap
1	606	Chr00 = 5, Chr14 = 1	317.16	7.48
2	484	–	124.55	4.74
3	508	–	179.57	7.23
4	593	–	240.75	11.31
5	407	–	129.64	3.83
6	299	Chr00 = 5, Chr07 = 1, Chr13 = 1	107.48	5.40
7	327	Chr00 = 3	184.58	8.42
8	198	Chr11 = 1	82.73	6.89
9	533	–	121.42	3.86
10	418	Chr00 = 2, Chr06 = 1, Chr15 = 1	145.60	4.18
11	395	Chr01 = 1, Chr15 = 4	178.53	5.52
12	573	Chr00 = 1, Chr09 = 2, Chr13 = 1, Chr15 = 1	178.08	6.58
13	324	–	213.23	11.16
14	336	Chr08 = 1	144.04	5.99
15	409	–	93.11	4.24
Total	6410	32	2440.47	–

**FIGURE 5:**
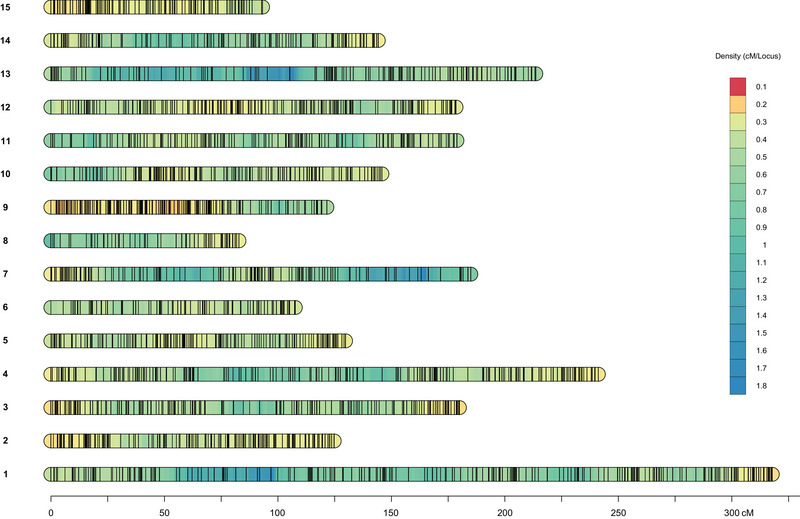
Linkage map displaying marker density across all chromosomes. Black lines represent marker positions, while color gradients indicate marker density. The linkage group length is depicted in centiMorgans.

As 54 SNPs were not placed along the map construction, the final genetic map consisted of 6410 SNPs distributed across 15 chromosomes, with the number of markers per chromosome ranging from 198 to 606. Chromosome sizes ranged from 82.73 (chromosome 8) to 317.16 cM (chromosome 1), resulting in a total map size of 2440.47 cM. The maximum gap between markers ranged from 3.86 to 11.31 cM (Table 2, Figure 5).

One of the first genetic linkage studies in *I. trifida* was conducted by Tomita et al. (2004) while mapping the self‐incompatibility locus (*S*‐locus) in the species. Eight markers linked to the *S*‐locus gave origin to a single LG of ∼23 cM where the *S*‐locus was contained within 1.25 cM. Later, an F_1_ (first filial generation) population derived from the cross between the 0431‐1 and Mx23‐4 was analyzed (Nakayama et al., 2010) using the pseudo‐testcross approach (Grattapaglia & Sederoff, 1994). The maternal map consisted of 618 markers distributed across 17 LGs, covering a total map length of 910 cM, while the paternal map was comprised of 163 markers distributed across 15 LGs, with a total length of 503.3 cM (Nakayama et al., 2010).

Finally, a more recent study evaluated the DSP4653‐21‐13 × DSP4597‐10‐21 F_1_ population of 238 individuals (Zhao et al., 2021). The maternal linkage map consisted of 904 markers distributed across 13 LGs, spanning 842.34 cM, while the paternal map contained 2034 markers distributed across 15 LGs, covering 1540.98 cM. An integrated genetic map included 2892 SNPs distributed across 15 LGs, with a total map length of 1550.22 cM (i.e., 1.32 SNPs per cM). Although greater in size, our integrated linkage map has more markers and is denser (2.63 SNPs per cM) than the previous one, making it suitable for refined QTL analysis.

### 3.3 QTL analyses

**FIGURE 6:**
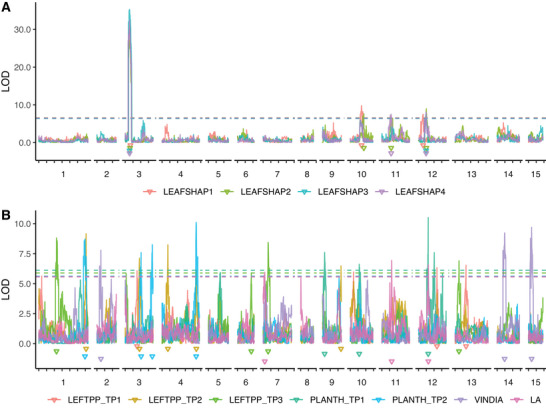
Logarithm of the odds (LOD) score profile from composite interval mapping of traits (A) leaf general outline (LEAFSHAP1), leaf lobes type (LEAFSHAP2), leaf lobe number (LEAFSHAP3), and leaf central lobe shape (LEAFSHAP4. (B) Number of leaves per plant measured at 12 days (LEFTPP_TP1), 30 days (LEFTPP_TP2), and 58–62 days (LEFTPP_TP3); plant height measured at 11 days (PLANTH_TP1) and 30 days (PLANTH_TP2); vine internode diameter (VINDIA); and leaf area (LA). Each triangle represents the QTL position according to thresholds (dashed lines) obtained from 1000 permutations. Ticks (*x*‐axis) are distributed every 50 cM along linkage groups.

The results of the CIM for each evaluated trait revealed a total of 37 QTLs. Two QTLs were identified for LEAFSHAP3; three for LEAFSHAP1, LEAFSHAP4, LEFTPP_TP1, PLANTH_TP1, VINDIA, and LA each; four for LEAFSHAP2, LEFTPP_TP3, and PLANTH_TP2 each; and five for LEFTPP_TP2 (Figure 6).

The QTLs were distributed across the 15 LGs, with eight QTLs identified on chromosome 3. Additionally, one QTL was identified on chromosomes 2, 6, 14, and 15 each; two QTLs were found on chromosomes 7, 9, and 13 each; three QTLs were detected on chromosomes 1, 4, 10, and 11 each; and seven QTLs were located on chromosome 12. No QTL was detected on chromosomes 5 and 8. For a logarithm of the odds (LOD) score > 3.0, 20 significant additive effects were attributed to the M9 parent, 14 to the M19 parent, whereas 13 significant dominance effects were observed. The proportion of phenotypic variance explained by each QTL (R^2^) reached up to 42.40% for the LEAFSHAP3 QTL on chromosome 3 (Table 3).

**TABLE 3:** Quantitative trait loci (QTLs) identified through composite interval mapping analyses for morphological traits in a full‐sib progeny of *Ipomoea trifida* from the M9 × M19 full‐sib population.

Trait	QTL	Marker	LG	Position (cM)	Global LOD	$R^{2}\;$(%)	Additive effect M9		Additive effect M19		Dominance effect	
							Estimate	LOD	Estimate	LOD	Estimate	LOD
Leaf general outline (LEAFSHAP1)	1	Chr03_2634116	3	33.45	31.07	36.32	−0.304	18.11	0.305	17.49	−0.120	3.92
	2	Chr10_18078811	10	72.28	9.77	5.47	−0.041	0.46	−0.091	2.06	0.189	7.34
	3	Chr12_1378180	12	29.80	7.48	9.40	0.228	3.52	0.076	0.61	−0.114	3.05
Leaf lobes type (LEAFSHAP2)	1	Chr03_2347218	3	31.01	28.05	39.88	−0.486	13.53	0.580	18.55	−0.052	0.21
	2	Chr10_19597490	10	87.36	7.63	4.76	−0.221	1.97	−0.133	1.35	0.256	3.68
	3	Chr11_2819666	11	59.95	7.03	4.40	0.197	1.84	−0.322	5.36	0.033	0.07
	4	Chr12_3168336	12	50.38	8.97	8.11	0.237	3.82	−0.226	3.41	−0.125	1.10
Leaf lobe number (LEAFSHAP3)	1	Chr03_2204303	3	28.00	35.29	42.40	−0.274	19.4	0.279	18.8	−0.044	0.61
	2	Chr12_2787339	12	48.84	7.82	9.46	0.103	3.30	−0.093	2.9	−0.091	2.65
Leaf central lobe shape (LEAFSHAP4)	1	Chr03_2204303	3	28.00	32.44	39.11	−0.501	16.98	0.541	18.88	−0.018	0.03
	2	Chr11_2819609	11	59.95	2.06	3.45	−0.033	0.02	−0.218	1.64	0.073	0.43
	3	Chr12_2787339	12	48.84	8.05	8.76	0.372	5.39	−0.036	0.07	−0.179	2.57
Number of leaves per plant measured at 12 days (LEFTPP_TP1)	1	Chr03_7783965	3	75.90	6.09	8.94	0.143	1.57	−0.055	0.22	−0.223	3.78
	2	Chr12_20760521	12	118.86	6.34	11.05	−0.264	5.49	0.013	0.01	0.087	0.64
	3	Chr13_9041151	13	70.55	6.54	8.29	0.252	5.26	−0.091	0.68	0.127	1.28
Number of leaves per plant measured at 30 days (LEFTPP_TP2)	1	Chr01_30410817	1	304.84	9.16	8.51	−0.383	5.49	−0.123	0.57	0.309	3.64
	2	Chr03_27381031	3	91.79	7.14	5.48	0.413	3.52	0.001	0.00	−0.487	4.87
	3	Chr04_3045638	4	37.48	8.24	8.50	−0.207	1.62	0.071	0.18	−0.417	5.93
	4	Chr04_29408368	4	221.00	6.13	9.44	−0.273	2.45	−0.152	0.87	0.284	2.65
	5	Chr09_22844916	9	119.21	6.49	7.00	0.310	3.45	0.243	2.04	−0.264	2.54
Number of leaves per plant measured at 58–62 days (LEFTPP_TP3)	1	Chr01_11083046	1	114.05	8.84	8.25	−4.926	4.60	0.644	0.12	2.299	2.23
	2	Chr06_23470071	6	89.14	5.91	7.10	−2.652	2.40	0.966	0.35	2.922	3.03
	3	Chr07_3604826	7	32.46	8.44	10.07	2.734	3.14	3.700	5.56	0.461	0.09
	4	Chr13_1910711	13	25.10	6.91	8.24	−0.092	0.00	2.862	2.65	−3.668	5.39
Plant height measured at 11 days (PLANTH_TP1)	1	Chr09_1515605	9	13.67	7.61	7.06	1.279	7.52	−0.252	0.33	−0.229	0.29
	2	Chr10_16210815	10	57.30	6.63	9.41	−0.851	3.46	0.807	3.33	−0.138	0.10
	3	Chr12_4116525	12	62.69	10.52	9.28	−0.727	1.01	−0.778	3.06	1.533	6.74
Plant height measured at 30 days (PLANTH_TP2)	1	Chr01_29840645	1	297.83	8.73	8.31	−4.660	6.93	1.715	1.06	−2.099	1.49
	2	Chr03_24395656	3	102.34	7.59	9.95	2.512	2.21	0.419	0.06	−4.105	5.58
	3	Chr03_12011722	3	174.36	8.25	8.47	1.883	1.23	4.495	6.65	−1.905	1.22
	4	Chr04_29345158	4	219.90	10.19	8.94	−9.322	7.22	−3.329	4.34	0.003	0.00
Vine internode diameter (VINDIA)	1	Chr02_8618789	2	25.39	7.79	6.84	0.000	0.01	0.016	7.18	−0.007	1.22
	2	Chr14_4027713	14	48.00	9.24	8.28	−0.010	2.69	−0.004	0.52	0.016	6.68
	3	Chr15_2674751	15	20.51	9.70	16.58	−0.017	7.89	0.008	1.76	0.003	0.25
Leaf area (LA)	1	Chr07_1838294	7	11.00	1.48	6.88	93.476	1.05	−70.761	0.17	−49.758	0.31
	2	Chr11_2832259	11	63.34	7.02	3.84	155.881	1.08	416.382	5.15	43.783	0.12
	3	Chr12_4356601	12	63.45	6.58	10.96	67.395	0.55	192.600	4.33	137.892	2.38

*Note*: It includes the proportion of phenotypic variance explained by each QTL (*R* ^2^), in percentage, along with the global and effect‐specific logarithm of the odds (LOD) ratio scores for each QTL.

Abbreviation: Chr, chromosome; LG, linkage group.

QTL mapping for *I. batatas* is limited, and many of the studies that aim to map QTL in the species are focused on starch and β‐carotene content, traits related to yield, and resistance to some types of pathogens, such as viruses and nematodes (da Silva Pereira et al., 2020; Fraher et al., 2024; Haque et al., 2020; Ma et al., 2020; Oloka et al., 2021; Sasai et al., 2019; Zheng et al., 2023). Even fewer studies have conducted QTL mapping in *I. trifida* (Suematsu et al., 2022; Zhao et al., 2021), and none have focused on morphological traits.

QTLs with notably high LOD scores were identified for leaf morphological traits on chromosome 3. Specifically, LEAFSHAP1, LEAFSHAP2, LEAFSHAP3, and LEAFSHAP4 exhibited LOD scores of 31.07, 28.05, 35.29, and 32.44, respectively. These results indicate strong associations between these traits and the corresponding regions on chromosome 3. Additionally, leaf traits such as LEAFSHAP1, LEAFSHAP2, LEAFSHAP3, LEAFSHAP4, LEFTPP_TP1, and LA showed QTL on chromosome 12. Among all traits, the lowest LOD score observed was for LA (1.48).

QTL co‐localization was also observed on chromosome 11 for LEAFSHAP2 and LEAFSHAP4, as well as on chromosomes 3 and 12 for LEAFSHAP3 and LEAFSHAP4. These findings may provide insights into the strong correlations observed between these traits (Figure 2), which could be due to closely linked or, most likely, pleiotropic genes. Xiao et al. (2023) identified SNPs associated with LLN on chromosomes 2 and 12 in *I. batatas* using genome‐wide association studies, with a peak at 38,790,815 bp on chromosome 12, where a candidate gene (*IbYABBY1*) was also located. In our *I. trifida* population, we detected strong QTL for leaf shape traits (LEAFSHAP1–4: peak positions 1,378,180, 3,168,336, 2,787,339, and 2,787,339 bp, respectively) on chromosome 12, all located at the beginning of the chromosome.

### 3.4 Putative gene search

Major QTLs pointed the putative gene search to the following regions: Chr03 (2,198,001–2,767,000 bp), Chr10 (18,053,372–19,572,051 bp), Chr11 (2,535,001–3,104,000), and Chr12 (1,377,840–3,168,330 bp). The full list of genes within those regions is available as Supporting Information S1.

In various species, studies show that multiple families of genes and proteins play essential roles in regulating leaf morphology. In rice (*Oryza sativa* L.), for example, the *curling leaf 1* gene, which encodes an MYB protein, not only regulates leaf development but also influences grain yield, representing an important genetic resource for plant improvement (Guo et al., 2023). Complementarily, research on maize and *Arabidopsis thaliana* has demonstrated that MYB‐like transcription factors act repressively on the *KNOX* gene family, with alterations in their activity resulting in significant modifications in leaf shape and lobulation patterns (Theodoris et al., 2003). The annotation for the MYB protein domain appears in all four chromosomes where QTLs for leaf morphology were identified (i.e., chromosomes 3, 10, 11, and 12). The genes associated with this annotation are *itf03g04470* on chromosome 3; *itf10g16340*, *itf10g16350*, and *itf10g16860* on chromosome 10; *itf11g05010*, *itf11g05040*, *itf11g05050*, and *itf11g05060* on chromosome 11; and *itf12g04070*, *itf12g04080*, *itf12g04100*, and *itf12g04360* on chromosome 12.

In *A. thaliana*, the RING finger protein responsive to brassinosteroids, encoded by the *BRH1* gene, integrates hormonal signals that define leaf structure, with its overexpression resulting in more curled rosette leaves, highlighting its role in leaf shape modulation and, consequently, in plant adaptation and productivity (Wang et al., 2018). In our study, the annotation brassinosteroid‐responsive RING‐H2 was found exclusively on chromosome 10, associated with the gene *itf10g16670*.

Additionally, mutations in MSCS‐like genes, which regulate plastid size and shape, also affect leaf morphology (Luesse et al., 2015). The gene *itf03g03930*, annotated as MSCS‐like, is located at the beginning of the chromosome 3 region. Meanwhile, the activation of AGAMOUS‐like genes can accelerate floral development and modify leaf morphology (Adal et al., 2021). The AGAMOUS‐like annotation in our study was found on chromosome 10, associated with *itf10g15990*.

Furthermore, the *AtFBX92* gene in *A. thaliana*, which encodes an F‐box protein, acts as a repressor of leaf growth. Its overexpression leads to significantly smaller leaves, whereas its downregulation stimulates cell proliferation (Baute et al., 2017). In our study, genes with the F‐box family protein annotation were identified on chromosome 10 (*itf10g16990*) and chromosome 12 (*itf12g02510*).

Regarding the AINTEGUMENTA‐like family, the ectopic expression of the *EgAP2‐1* gene in oil palm (*Elaeis guineensis*) promotes changes in leaf morphology and regenerative capacity, impacting plant productivity and adaptation (Morcillo et al., 2007), and the identification of *MtANT* genes in *Medicago truncatula* reinforces their importance in leaf growth and architecture (Wang et al., 2022). In our study, the AINTEGUMENTA‐like annotation was found on chromosome 3, associated with *itf03g04010*. Moreover, research on moso bamboo (*Phyllostachys edulis*) demonstrated that NIN‐like proteins, initially associated with nitrate signaling, also influence cell division and differentiation, altering leaf size, shape, and structure (Lin et al., 2021). In our study, the NIN‐like protein annotation was identified on chromosome 10, associated with *itf10g16290*.

These findings highlight the complex regulatory network that integrates various internal and environmental signals to define leaf morphology, which is crucial for plant adaptation and productive performance. Zhu et al. (2016) and Hu et al. (2018) emphasize the critical role of leaf morphology in determining a plant's quality, yield, and adaptability. Bo et al. (2022) highlight examples such as zucchini (*Cucurbita pepo*), which benefits from its lobed leaves, making it well‐suited for high‐density planting and large‐scale production. Similarly, Hu et al. (2018) noted the advantages of leaf traits in rapeseed (*Brassica napus*), a key crop for biodiesel production. These examples underscore the importance of analyzing leaf traits to develop cultivars optimized for human and animal consumption or even ornamental purposes.

## 4 FINAL CONSIDERATIONS

Leaves serve as valuable indicators of sweetpotato adaptability to various growing conditions and play a crucial role in the selection of crop varieties. In landscaping and ornamental uses, the most desirable traits include foliage coverage, leaf shape and size, flower and leaf color, as well as tolerance to insects and diseases (Abdallah, 2022; Jackson et al., 2020; de Sousa et al., 2018). The morphology‐related QTLs identified in our study could provide valuable insights for future research focused on enhancing ornamental sweetpotato varieties.

Comparative mapping approaches have been successfully applied to investigate morphological traits in other species, such as cotton and apple (Jiang et al., 2000; Xue et al., 2017). To validate the markers linked to the QTL identified in this study for hexaploid sweetpotato, a targeted approach could involve identifying the genes within the hexaploid plant sequence and genotyping a population of the species to assess the information transferability. Additionally, marker‐assisted selection in other segregating populations or genetic diversity panels, followed by association studies, would help confirm the robustness of those linked markers across different environments, ensuring their reliability for breeding applications.

## AUTHOR CONTRIBUTIONS

**Ana Paula Alves da Mata**: Data curation; formal analysis; investigation; methodology; visualization; writing—original draft. **Dorcus C. Gemenet**: Conceptualization; data curation; investigation; resources; writing—review and editing. **Federico Diaz**: Data curation; investigation; methodology; writing—review and editing. **Maria David**: Data curation; investigation; methodology; writing—review and editing. **Veronica Mosquera**: Data curation; investigation; methodology; writing—review and editing. **João Ricardo Bachega Feijó Rosa**: Formal analysis; investigation; methodology; writing—review and editing. **Iara Gonçalves dos Santos**: Data curation; formal analysis; methodology; writing—review and editing. **Gabriel de Siqueira Gesteira**: Data curation; formal analysis; investigation; methodology; writing—review and editing. **Marcelo Mollinari**: Formal analysis; methodology; writing—review and editing. **Awais Khan**: Conceptualization; funding acquisition; project administration; resources; supervision; writing—review and editing. **G. Craig Yencho**: Conceptualization; funding acquisition; project administration; resources; supervision; writing—review and editing. **Zhao‐Bang Zeng**: Funding acquisition; project administration; supervision; writing—review and editing. **Guilherme da Silva Pereira**: Conceptualization; data curation; formal analysis; investigation; methodology; project administration; supervision; visualization; writing—review and editing.

## CONFLICT OF INTEREST STATEMENT

The authors declare no conflicts of interest.

## Supporting information



Supplementary Material

Supplementary Material

**Figure S1**. Parents of M9 and M19, (A) CIP 460410 (DLP 4653), and (B) CIP460377 (DLP4597), showing overall shoot (top) and root (bottom) morphology.
**Figure S2**. Descriptors of morphological leaf shape‐related traits in sweetpotato. Source: CIP, AVRDC, IBPGR (1991).
**Figure S3**. Samples of leaves from M9 (A) and M19 (B).
**Figure S4**. Grouping and ordering based on genome information. From left to right, top to bottom, chromosomes 1 to 15.
**Figure S5**. Grouping based on UPGMA and ordering based on the reference genome. From left to right, top to bottom, chromosomes 1 to 15.
**Figure S6**. Final marker grouping and ordering. From left to right, top to bottom, chromosomes 1 to 15.

## Data Availability

The datasets analyzed in this study can be found on Github (https://github.com/ufv‐molecular‐breeding‐lab/amata_itrifida_linkage_mapping). The variant call formats are available at the Dryad repository (https://doi.org/10.5061/dryad.vx0k6dk4w).
