# Supplementary material for: Linkage map construction and QTL mapping for morphological traits in Ipomoea trifida, a diploid sweetpotato relative
Source: Plant Genome. 2025 Sep 18;18(3):e70106. doi: 10.1002/tpg2.70106 (PMC12445336; doi:10.1002/tpg2.70106)
Supplement: Supplementary file 1 [file TPG2-18-e70106-s001.docx]

# 1  Supplementary figures

Figure S1. Parents of M9 and M19, (A) CIP 460410 (DLP 4653), and (B) CIP460377 (DLP4597), showing overall shoot (top) and root (bottom) morphology.

Figure S2. Descriptors of morphological leaf shape related traits in sweetpotato. Source: CIP et al. (1991).

Figure S3. Samples of leaves from M9 (A) and M19 (B).

**Figure S4.** Grouping and ordering based on genome information. From left to right, top to bottom, chromosomes 1 to 15.

**Figure S5.** Grouping based on UPGMA and ordering based on the reference genome. From left to right, top to bottom, chromosomes 1 to 15.

**Figure S6.** Final marker grouping and ordering. From left to right, top to bottom, chromosomes 1 to 15.
